# Cigarette smoke extract induces differential expression levels of beta-defensin peptides in human alveolar epithelial cells

**DOI:** 10.1186/1617-9625-11-10

**Published:** 2013-04-29

**Authors:** Tony Pierson, Sarah Learmonth-Pierson, Daniel Pinto, Monique L van Hoek

**Affiliations:** 1 School of Systems Biology, George Mason University, Manassas, VA, USA; 2 National Center for Biodefense and Infectious Diseases, George Mason University, Manassas, VA, USA; 3 Discovery Hall, George Mason University, 10910 University Blvd, MS1H8, Manassas, VA, 20120, USA

**Keywords:** Cigarette smoke extract, β-defensins, Lung, Alveolar epithelial cells, Antimicrobial peptide

## Abstract

**Background:**

The damaging effects of cigarette smoke on the lungs are well known in terms of cancer risks. Additional molecular changes within the lung tissue can also occur as a result of exposure to cigarette smoke. The human β-defensin (hBD) class of antimicrobial peptides is the focus of our research. In addition to antimicrobial activity, β-defensins also have immunomodulatory functions. Over 30 previously unrecognized β-defensin genes have recently been identified in the human genome, many with yet to be determined functions. We postulated that altered β-defensin production may play a role in the pathogenesis observed in the lungs of smokers. Our hypothesis is that cigarette smoke exposure will affect the expression of β-defensins in human lung alveolar epithelial cells (A549).

**Methods:**

We exposed A549 cells to cigarette smoke extract (CSE) and measured the changes in mRNA levels of several antimicrobial peptides by quantitative real-time PCR, and directly observed peptide expression in cells by immunofluorescence (IF) microscopy.

**Results:**

We found that hBD3, hBD5, and hBD9 gene expression was upregulated in A549 cells exposed to CSE. HBD1, hBD8, hBD18 and LL-37 gene expression did not significantly change upon exposure to CSE. Expression of hBD3 and hBD4 peptides was visualized by IF.

**Conclusions:**

This differential expression suggests that hBD3, hBD5, and hBD9 may play a role in the changes to the lung tissue observed in smokers. Establishing differential β-defensin expression following CSE treatment will add to our understanding of the molecular response of the lung alveolar epithelium to cigarette smoke exposure.

## Background

Smoking cigarettes increases the risk of contracting lung cancer [1]. Lung cancer caused an estimated 159,300 deaths in the United States in 2009, and is the leading cause of cancer related mortality [2]. In addition to causing cancer, smoking increases the risk of dying prematurely due to chronic obstructive pulmonary disease (COPD) or emphysema [3], stroke and cardiac arrest [4]. Cigarette smoke contains thousands of chemical components, many of which are capable of acting as either carcinogens [5] or foreign antigens [6-8]. Once inhaled, some cigarette smoke components adhere to cells lining the respiratory tract, triggering a robust response by lung cells, including activating the innate immune system [9].

Antimicrobial Peptides (AMPs) are small (<100 amino acids) peptides that play a critical role in host airway defense as part of the innate immune system [10]. α and β are the two major classes of defensin AMPs. β-defensin expression is associated with some chronic lung diseases, including COPD [11-14]. In addition to α and β defensins, humans possess one member of the cathelicidin class of AMP, LL-37, which was also assessed in these experiments. α-defensins are not expressed in epithelial cells, and were not measured in the present study.

The Human Lung Alveolar Epithelial (A549) cell line is a model system used to model alveolar epithelial type II cells (ATII). We used this model for the quantification of relative AMP expression levels after exposure to bacteria [15]. Although this model has been extensively used in the literature, the limitation of this model is that it does not undergo differentiation to express the alveolar type I (ATI) phenotype [16]. Given the specific goal of this experiment, we determined that the A549 model was an appropriate tool for our study. In this experiment we tested mRNA level changes in response to exposure to cigarette-smoke extract (CSE) of known as well as recently identified β-defensin genes [17-20].

Until recently, only four human β-defensins (hBDs) were known to be expressed in respiratory epithelial cells (hBD1, hBD2, hBD3 and hBD4) [21-23]. However, recent computational and genomic research has predicted over 30 additional human β-defensins, many of which have not been investigated for their biological function [17,24]. Importantly, defensin peptides appear to play multiple roles *in vivo* and exhibit an immunomodulatory function, similar to cytokines or chemokines. Expression of β-defensins can be induced via NF-κB activation mainly through TLR receptor binding to microbial components, or via pro-inflammatory cytokines, such as TNF-α and IL1-β. Weinberg characterized β-defensins as complex molecules whose proper function is critical for effective immune system function and that when β-defensin production is altered, especially in areas where cells are already not functioning properly, AMP expression may actually increase disease progression [25]. In addition to the pathological changes observed in lungs exposed to smoke, exposed bronchial epithelial cells secrete many cytokines with the ability to stimulate immune cell migration [26]. β-defensin expression may also produce a cellular environment where inflammation is enhanced [25] and vascular permeability is increased [27].

Recent publications have shown that exposure of cells to cigarette smoke represses the induction of hBD2 expression by cells exposed to the bacterial lipopolysaccharide [28,29]. Human gingival epithelial cells exposed to cigarette smoke show increased expression of several β-defensins as well as proinflammatory cytokines [30]. Cigarette smoke directly or indirectly alters the expression of hBD2 [31]. It has also been suggested that this peptide may play a role in the pathogenic effects caused by cigarette smoke [20]. Our hypothesis is that exposure of Human Alveolar Epithelial Cells (A549) to cigarette smoke will lead to measurable changes in gene expression levels for some of the newly-identified human β-defensin peptides (hBDs) as measured by quantitative real-time PCR (qRT-PCR). Our finding that exposure to cigarette smoke increases certain β-defensin gene expression would suggest that repeated exposure with each new cigarette would lead to chronic over-expression of those β-defensins and possibly exacerbate the deleterious effects of cigarette smoke-damaged cells.

## Materials and methods

### β-defensin Induction in A549 cells

Low passage human lung epithelial cell line (A549, ATCC CCL-185) were grown to 95% confluence in 6 well culture plates (1 × 10^6^ cells per well, BD catalog # 353046) with Ham’s F-12 (Cellgro 10-080-CV) containing 10% heat inactivated fetal bovine serum, 37°C, 5% CO_2._ Cells were then washed with PBS and fed serum-free Hams F-12 media overnight. Following the previously established protocol [5,31], cells were exposed to either 50 μg/ml of the 40 mg/ml stock solution of cigarette smoke extract (CSE in 100% DMSO) in Ham’s F-12 media, or an equivalent amount of DMSO alone in Ham’s F-12 media, or 10 ng/ml of IL-1β (Sigma) in Ham’s F-12 media, for 30 minutes.

### Cigarette Smoke Extract (CSE)/Cigarette smoke condensate (CSC)

CSE was purchased from Murty Pharmaceuticals, Lexington KY [5,31]. According to the manufacturer CSE was produced by burning one University of Kentucky “1R13” cigarette and extracting the total particulate into 100% DMSO to prepare a 40 mg/ml stock solution. Cells were exposed to 50 μg/ml of the 40 mg/ml stock solution of cigarette smoke extract (CSE in 100% DMSO) suspended in Ham’s F-12 media. The control cells received the equivalent volume of DMSO devoid of CSE suspended in Ham’s F-12 media. Previously published reports using this material were followed [5,31].

### Analysis of AMP gene expression by quantitative reverse transcription qRT-PCR

After exposure, cells were harvested, mRNA, extracted, and cDNA made following our previously published protocol for qRT-PCR of antimicrobial peptides [15,32]. Quantitative RT-PCR was performed with primers specific for the following β-defensin peptides: hBD1, hBD3, hBD5, hBD6, hBD8, hBD9, hBD18, and the cathelicidin LL-37 in order to measure how much each gene was activated or inhibited by the cigarette smoke treatment. Control cells were used as a baseline to compare induction of gene expression in cells exposed to cigarette smoke extract, and levels of 18S rRNA were used to normalize between samples, leading to the relative units of expression indicated for each experiment. Using a previously established protocol [15,32], quantitative real-time PCR analysis in a MyiQ Single Color Real-Time PCR Detection System (BioRad Laboratories) was performed according to the manufacturer’s instructions. Primer sequences and melting temperatures are summarized in Table 1.

**Table 1:** Quantitative RT-PCR primer sequences used in this study

**Primers**	**Sequences**	**Melting temp**	**Product size**	**Reference**
HBD1 Forward	5′-CCCAGTTCCTGAAATCCTGA-3′	56°C	215bp	(Han et al., 2008) [15]
Reverse	5′-CAGGTGCCTTGAATTTTGGT-3′	56°C		
HBD3 Forward	5′-AGCCTAGCAGCTATGAGGATC-3′	56°C	206bp	(Han et al., 2008) [15]
Reverse	5′-CTTCGGCAGCATTTTGCGCCA-3′			
HBD5 Forward	5′-TCCATCAGGTGAGTTTGCTG-3′	57°C	105bp	(*)
Reverse	5′-GTTCAGCCTGCAATTTCCAT-3′			
HBD8 Forward	5′-TGCCTTGAAACAGAAATCCA-3′	56°C	104bp	(**)
Reverse	5′-TCCTTTTTGGGTGTAGTGCTC -3′			
HBD9 Forward	5′-GGCCTAAATCCAGGTGTGAA-3′	57°C	174bp	(Alekseeva et al., 2009) [33]
Reverse	5′-TCAAATGTTGGCAAGTGGAG-3′			
HBD18 Forward	5'-TGCATTCCATCCAATGAAGA-3′	57°C	181bp	(***)
Reverse	5′-GAGGTCTCAGTTCCCCTTCC-3′			
LL-37 Forward	5′-CTAGAGGGAGGCAGACATGG-3′	57°C	201bp	(Amer, Bishop, & van Hoek, 2010) [32]
Reverse	5′-AGGAGGCGGTAAGGTTAGC-3′			

This table summarizes the primers that were used in this study, the melting temperature, the predicted product size and relevant references. Invitrogen OligoPerfect™ Designer was used to design new primers. (*) NM_152250.1 Homo sapiens defensin, beta 105A (DEFB105A), mRNA; (**) NM_001002035.1 Homo sapiens defensin, beta 108B (DEFB108B), mRNA; (***) NM_054112.2 Homo sapiens defensin, beta 118 (DEFB118), mRNA.

### Immunofluorescence

A549 cells were grown on chambered slides (BD Falcon, 354108) and exposed to CSE as above for β-defensin induction. Cells were fixed, permeabilized and blocked as previously described [34], and a 1:500 dilution of anti-human β-defensin primary antibody (Abcam, ab14421 Anti-beta Defensin 1 antibody, ab19270 Anti-beta 3 Defensin antibody, ab70215 Anti-beta 4 Defensin antibody) was applied for 1 hr. Detection was performed with 1:5000 dilution of secondary antibody labeled with Alexafluor 488 (Green), using ProLong Gold anti-fade with DAPI (Blue). Images were obtained as previously described [34], using the Nikon Confocal microscope.

### Statistical analysis

Statistical analysis and graphing was carried out using Prism 5 software (GraphPad, La Jolla, CA). The two-tailed t-test assuming unequal variance was used to compare negative control β-defensin gene expression of the two different experimental groups expression profiles (IL-1β, cigarette smoke extract, CSE) compared to the control (DMSO alone).

### Ethical & safety approvals

All experiments were approved by the Institutional Review Board at George Mason University. Since only a commercially available, de-identified human cell line was used, no ethical approvals were required for this research.

## Results

Based on emerging reports of potentially important roles for some of the newly discovered human β-defensins in lung defense (hBD3, hBD5, hBD8, hBD9, hBD18) [18,24,33], and the emerging role of hBD1 in COPD [11], we chose to study this selected set of the human β-defensin genes that had not been previously examined for their alteration following exposure to cigarette smoke (CSE) [29,35]. We included the human cathelicidin, LL-37/CAMP, as a comparator, as it also is thought to play a significant role in lung defense [17].

### HBD1

**Figure 1:**
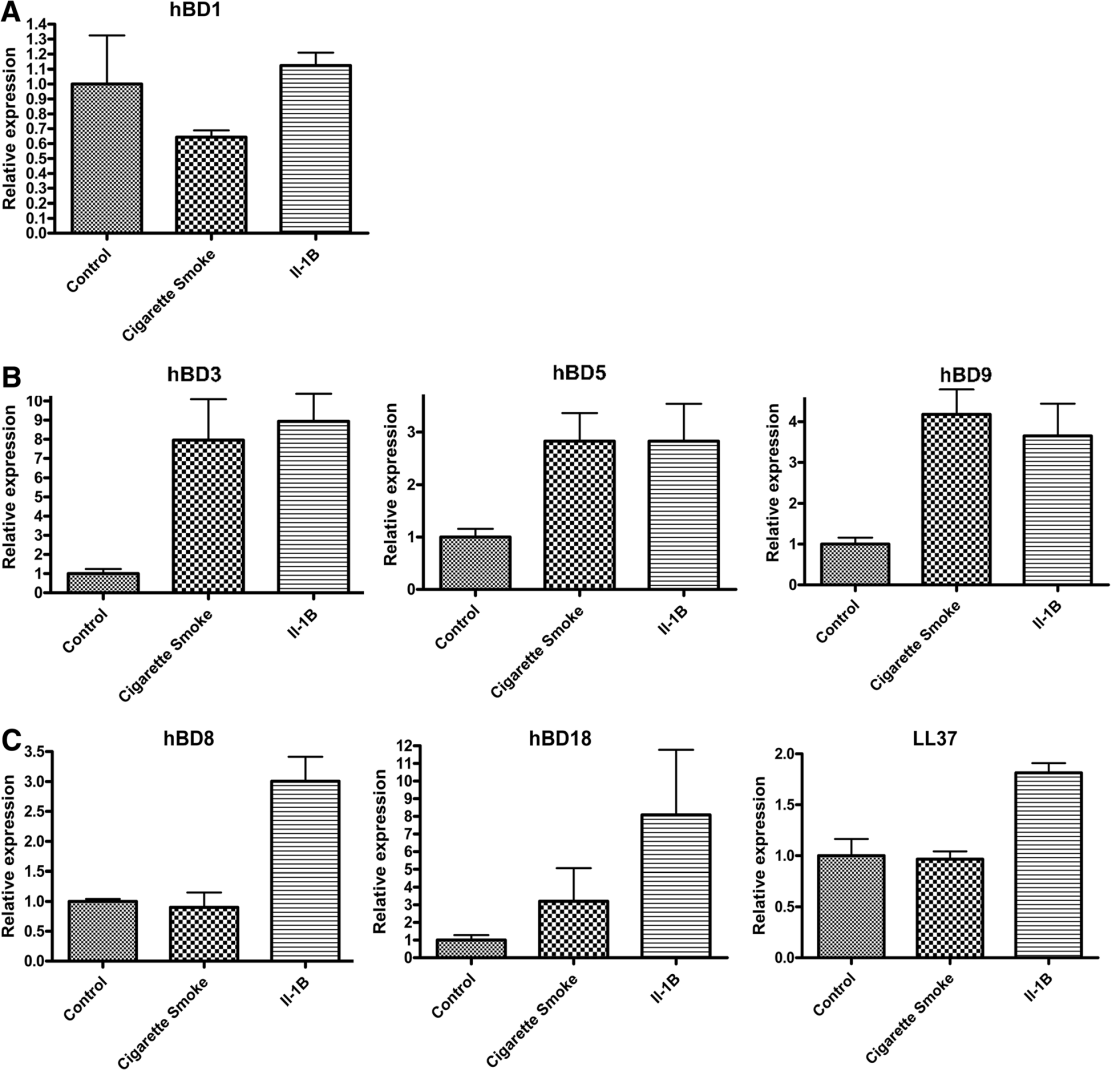
**Expression of Antimicrobial peptides A. Expression of hBD1.** The constitutively expressed β-defensin, hBD1, is not induced by IL-1β treatment, as measured by qRT-PCR (Control = 1.000+/−0.324, IL-1β =1.123+/−0.086) (n = 3). Although the means differ, the treatment by cigarette smoke extract (CSE) did not statistically significantly alter the level of hBD1 expression as measured by T-test (Control = 1.000+/−0.324, CS = 0.643+/−0.046, p > 0.05). **B**. Expression of hBD3, hBD5, and hBD9. Cigarette Smoke Extract (CSE) induces the expression of hBD3, hBD5 and hBD9. Following treatment with CSE the expression of hBD3, hBD5 and hBD9 were all significantly (p < 0.05) induced as measured by qRT-PCR to levels similar to those achieved with IL-1β treatment. (hBD3 was 7.95 fold increased (p = 0.032), hBD5 was 2.8 fold increased (p = 0.0083) and hBD9 was 4.2 fold increased (p = 0.0078). n = 3 for hBD3, 9, n = 6 for hBD5). **C**. Expression of hBD 8, hBD18 and LL-37. Cigarette Smoke Extract (CSE) does NOT induce expression of hBD8, hBD18, LL-37. Measured by qRT-PCR, the expression of hBD8 and LL-37 is not significantly induced in response to cigarette smoke extract (p > 0.05), however these defensins are significantly (p < 0.05) induced by IL-1β treatment. HBD18 was also not statistically significantly induced with cigarette smoke extract, although the means differed (Control = 1.000+/−0.287, CSE = 3.207+/−1.862, p = 0.31)(n = 3).

The first identified human β-defensin gene, hBD1, is considered a constitutively produced antimicrobial peptide, whose expression has not been observed to change significantly under physiological conditions [36,37]. Following treatment with 50 μg/ml cigarette smoke (CSE), there is no significant change in hBD1 expression (Control = 1.000+/−0.324, CSE = 0.643+/−0.046, p > 0.05) compared to mock treated cells. This confirms previous findings, as the expression of this peptide is also not up-regulated by 10 ng/ml of IL-1β treatment (IL-1β =1.123+/−0.086) (n = 3) compared to mock treated cells (Figure 1A).

### HBD3, hBD5 and hBD9

The human β-defensins hBD3, hBD5 and hBD9 were found to be significantly increased following treatment with 50 μg/ml cigarette smoke extract (CSE) when compared to mock treated cells, and induced as much as with 10 ng/ml of IL-1β treatment. In response to CSE, hBD3 was 7.95 fold increased (p = 0.032), hBD5 was 2.8 fold increased (p = 0.0083) and hBD9 was 4.2 fold increased (p = 0.0078). (n = 3 for hBD3, 9, n = 6 for hBD5) (Figure 1B).

### HBD8 and hBD18

Expression of the antimicrobial peptide gene hBD8 was not significantly induced in response to 50 μg/ml cigarette smoke extract (CSE) (p > 0.05), however this β-defensin is significantly (p < 0.05) induced by 10 ng/ml of IL-1β treatment. The expression of another β-defensin peptide gene hBD18 was not statistically significantly induced with 50 μg/ml cigarette smoke extract, although the means differed (Control = 1.000+/−0.287, CSE = 3.207+/−1.862) (p = 0.31). (Figure 1C).

### LL-37

Expression of the cathelicidin gene LL-37 was not significantly induced in response to 50 μg/ml cigarette smoke extract (CSE) (p > 0.05) either, however this antimicrobial peptide gene is significantly (p < 0.05) induced by 10 ng/ml of IL-1β treatment. (Figure 1C).

### Visualization of hBDs by fluorescent microscopy

**Figure 2:**
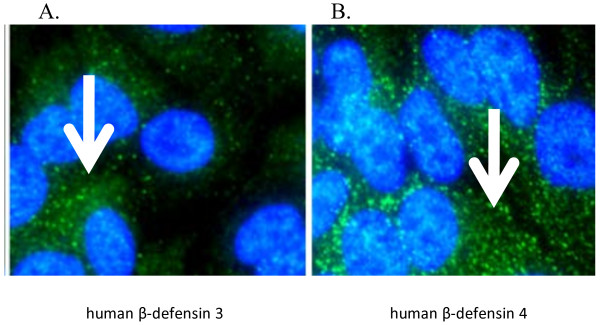
**Immunofluorescence Microscopy of hBDs in A549 cells treated with cigarette smoke extract.** Green = human β-defensin (Alexafluor 488), Blue = nuclei (DAPI). **A**. hBD3 protein is observed in punctate staining in A549 cells (White arrow points to punctate staining). **B**. hBD4 protein is observed in heavily punctate staining in A549 cells (White arrow points to punctate staining).

A549 cells exposed to 50 μg/ml cigarette smoke (CSE) and processed *in situ* displayed positive staining for the internal presence of human β-defensins (Figure 2). Immunofluorescent microscopy was performed of cigarette-smoke treated A549 cells staining for hBD1, hBD3, and hBD4. Shown are representative images of staining for hBD3 and hBD4 peptides. No differential defensin staining was observed in the cells for hBD1 (data not shown), suggesting a similar result as was shown by the lack of induction of hBD1 seen in the qRT-PCR study.

## Discussion

A549 cells are considered to be a cell-line model for Type II alveolar epithelial cells, which secrete the lung surfactant and A549 express some of the markers of Type II cells [38,39]. In this study, we use the A549 cell model to measure relative gene expression of two classes of AMP; human β-defensins (hBDs) and the human cathelicidin (LL-37) in response to exposure to cigarette smoke extract. Total RNA was extracted from the samples and quantitative real-time PCR was performed to quantify AMP gene expression using gene specific primers.

The experimental approach was to expose A549 cells to 50 μg/ml cigarette smoke extract (dissolved in DMSO), compared to control cells treated with DMSO alone. The positive control cells were exposed to 10 ng/ml IL-1β which is known to up-regulate the expression of many defensin peptides [33]. The mRNA was then prepared from all samples, and qRT-PCR was performed for hBD1, hBD3, hBD5, hBD8, hBD9, hBd18, and the Cathelicidin LL-37. Overall, the application of 10 ng/ml IL-1β to A549 cells induced the expression of most of the tested human β-defensins (except hBD1, see below), confirming that the cells responded to the stimuli as expected.

We found that hBD3, hBD5, and hBD9 gene expression was up-regulated in A549 cells exposed to 50 μg/ml CSE, while hBD1, hBD8, hBD18 and LL-37 gene expression did not significantly change. Expression of hBD1, hBD3 and hBD4 peptides within the A549 cells (*in situ*) was visualized by IF. This differential expression suggests that hBD3, hBD5, and hBD9 may play a role in the changes to the lung tissue observed in smokers. Establishing the differential human β-defensin gene expression following CSE treatment will add to our understanding of the molecular response of the lung alveolar epithelium to cigarette smoke exposure.

Our data demonstrates the differential induction of expression of specific β-defensin peptide genes as a result of exposure to cigarette smoke extract and suggests that three β-defensin genes (hBD3, hBD5, hBD9) may represent biomarkers of cigarette smoke extract exposure for alveolar epithelial cells [40-46]. These findings may contribute to a greater understanding of the role of the newly identified β-defensin peptides in the response of lung epithelial cells to cigarette-smoke. Further studies of other sample types from smokers, such as bronchoalveolar lavage could be used to validate these findings. Such biomarkers may have utility in studying the recovery of lung cells following the cessation of smoking.

Gene expression for hBD1 was not changed by exposure to cigarette smoke or IL-1β, as expected. The expression of hBD5, hBD8, hBD9 and hBD18, four of the new members of the human β-defensin gene family, are inducible by IL-1β in A549 lung epithelial cells, indicating they may respond to IL-1β regulation *in vivo* and may be involved in inflammatory responses and cell proliferation, differentiation, and apoptosis. Three genes, hBD8, hBD18 and the cathelicidin LL-37, were induced with IL-1β treatment, but not with cigarette smoke treatment, suggesting there is specificity of regulation for the different β-defensins. hBD3, hBD5, and hBD9 gene expression was increased by cigarette smoke extract treatment to the same extent as with IL-1β treatment. This suggests that these three β-defensins may have similar regulation by components of the cigarette smoke extract. Similarly, differentially-regulated expression of hBD3 and hBD9 was observed in infectious keratitis of the human eye [47] supporting that differential regulation of human β-defensin gene expression can occur. HBD3 is thought to be an important component of lung defense, such as in response to *Legionella* infection [48].

Exposure to cigarette smoke is thought to be harmful to host respiratory defenses through multiple modes of action [8]. Recent studies have suggested that cigarette smoke alters the production of hBD2 [28,29,49-54]. The induction of hBD2 and hBD3 expression (as well as IL-1B) in human gingival epithelial cells by cigarette smoke is thought to be through the activation of ERK1/2 and NF-kB pathways [30]. Nictotine has been reported to upregulate the expression of hBD2 via a p38 MAPK dependent pathway in human keratinocytes [55]. Cigarette smoke contains many toxic components, including potentially bacterial lipopolysaccharides [56], which in combination with the delivered nicotine could contribute to these observed effects of cigarette smoke. HBD2 is expressed in keratinocytes, the gingival mucosa and the tracheal epithelium [48,57-59] and alteration of its gene expression has been found in many diseases, such as infectious diseases, CF, and lupus erythematosus [60].

Xu recently showed that cigarette smoke extract significantly increased the production of IL-1β [57]. It was also shown that cigarette smoke inhibits the subsequent induction of hBD2 expression in response to bacterial lipopolysaccharide [52] or IL-1β [51], suggesting that cigarette smoke also may suppress the normal host defense response to bacterial pathogen exposure in the lung [35]. Kanda et al. recently demonstrated that exposure to hBD2 enhanced mRNA levels and secretion of many cytokines, including IL-1β [58]. Therefore, cigarette smoke may directly stimulate novel hBD production, leading to enhancement of IL-1β, which in turn could further stimulate additional hBD production, in a paracrine or autocrine loop. Interestingly, Kanda also reported that the effects of hBD2 were counteracted by the drug Pentoxifylline (PTX) [58]. PTX is a drug with strong anti-inflammatory properties. This observation suggests that we should investigate the ability of PTX to potentially counteract the effect of chronic CSE-induced over-expression of human β-defensins.

Stimulation of oral squamous cell carcinoma (BHY-OSCC) cell line with hBD1 results in reduction of cell proliferation, whereas hBD2 and hBD3 stimulation causes promotion of cell proliferation, indicating that hBD2 and hBD3 might be protooncogenes in OSCCs [59]. Recent studies demonstrated all lung tumor samples, independent of their histological type, express hBD2 peptide, and its expression levels correlates with the differentiation grade of lung adenocarcinoma [61], suggesting that the role of human β-defensins in lung cancer should be further investigated also.

The health effects of cigarette smoke on lung health are significant. Establishing a link between smoking and the induction of novel and established antimicrobial peptides is an important step in understanding the pathology observed in cigarette smokers. AMPs may represent novel biomarkers of overall lung cell health status, as well as having potential direct effects in the pathogenesis of cigarette smoking. In the long term, we would like to investigate whether chronic antimicrobial peptide induction may contribute to lung pathology, which may occur before the occurrence of cancer or Chronic Obstructive Pulmonary Disease (COPD). If so, AMP induction could potentially be useful as both a biomarker of cigarette exposure, as well as a possible target for therapeutic intervention.

## Abbreviations

CSE: Cigarette smoke extract; HBD: Human beta-defensin; BALF: Bronchiolar lavage fluid; TNF-α: Tumor necrosis factor alpha; TLR: Toll-like receptor; CF: Cystic fibrosis; COPD: Chronic obstructive pulmonary disease; PCR: Polymerase chain reaction; IF: Immunofluorescence; DMSO: Dimethly sulfoxide; mRNA: messenger ribonucleic acid; DAPI: 4′,6-diamidino-2-phenylindole; IL-1β: Interleukin-1 beta; LPS: Lipopolysaccharide.

## Competing interest

The authors declare that they have no competing interest.

## Authors’ contributions

MVH conceived and designed the study. TP helped to design the study, performed key experiments and did data analysis. MVH and TP co-wrote the manuscript. SLP and DP performed some qRT-PCR experiments and contributed to the data analysis. All authors read and approved the final manuscript.

## References

[B1] Doll R Uncovering the effects of smoking: historical perspective Stat Methods Med Res 1998 7 2 87 117 10.1191/096228098668199908 9654637

[B2] Sangodkar J Lung adenocarcinoma: lessons in translation from bench to bedside Mt Sinai J Med 2010 77 6 597 605 10.1002/msj.20226 21105123

[B3] Brusselle GG Joos GF Bracke KR New insights into the immunology of chronic obstructive pulmonary disease Lancet 2011 378 9795 1015 1026 10.1016/S0140-6736(11)60988-4 21907865

[B4] McMaster SK Cigarette smoke inhibits macrophage sensing of Gram-negative bacteria and lipopolysaccharide: relative roles of nicotine and oxidant stress Br J Pharmacol 2008 153 3 536 543 10.1038/sj.bjp.0707595 18059323 PMC2241791

[B5] Kier LD Yamasaki E Ames BN Detection of mutagenic activity in cigarette smoke condensates Proc Natl Acad Sci U S A 1974 71 10 4159 4163 10.1073/pnas.71.10.4159 4610572 PMC434349

[B6] Smith CJ IARC carcinogens reported in cigarette mainstream smoke and their calculated log P values Food Chem Toxicol 2003 41 6 807 817 10.1016/S0278-6915(03)00021-8 12738186

[B7] Shin HJ Effect of cigarette filters on the chemical composition and in vitro biological activity of cigarette mainstream smoke Food Chem Toxicol 2009 47 1 192 197 10.1016/j.fct.2008.10.028 19027817

[B8] Nikota JK Stampfli MR Cigarette smoke-induced inflammation and respiratory host defense: Insights from animal models Pulm Pharmacol Ther 2012 25 4 257 262 10.1016/j.pupt.2012.05.005 22634304

[B9] Dwyer TM Cigarette smoke-induced airway inflammation as sampled by the expired breath condensate Am J Med Sci 2003 326 4 174 178 10.1097/00000441-200310000-00004 14557729

[B10] Doss M Human defensins and LL-37 in mucosal immunity J Leukoc Biol 2010 87 1 79 92 10.1189/jlb.0609382 19808939 PMC7167086

[B11] Andresen E Increased expression of beta-defensin 1 (DEFB1) in chronic obstructive pulmonary disease PLoS One 2011 6 7 e21898 10.1371/journal.pone.0021898 21818276 PMC3139569

[B12] Liao Z Enhanced expression of human beta-defensin 2 in peripheral lungs of patients with chronic obstructive pulmonary disease Peptides 2012 38 2 350 356 10.1016/j.peptides.2012.09.013 23000304

[B13] Janssens W Genomic copy number determines functional expression of beta-defensin 2 in airway epithelial cells and associates with chronic obstructive pulmonary disease Am J Respir Crit Care Med 2010 182 2 163 169 10.1164/rccm.200905-0767OC 20378733

[B14] Matsushita I Genetic variants of human beta-defensin-1 and chronic obstructive pulmonary disease Biochem Biophys Res Commun 2002 291 1 17 22 10.1006/bbrc.2002.6395 11829455

[B15] Han S Bishop BM van Hoek ML Antimicrobial activity of human beta-defensins and induction by Francisella Biochem Biophys Res Commun 2008 371 4 670 674 10.1016/j.bbrc.2008.04.092 18452706

[B16] Swain RJ Assessment of cell line models of primary human cells by Raman spectral phenotyping Biophys J 2010 98 8 1703 1711 10.1016/j.bpj.2009.12.4289 20409492 PMC2856139

[B17] Hiemstra PS Defensins and cathelicidins in inflammatory lung disease: beyond antimicrobial activity Biochem Soc Trans 2006 34 Pt 2 276 278 16545093 10.1042/BST20060276

[B18] Schutte BC Discovery of five conserved beta -defensin gene clusters using a computational search strategy Proc Natl Acad Sci U S A 2002 99 4 2129 2133 10.1073/pnas.042692699 11854508 PMC122330

[B19] Rodriguez-Jimenez FJ Distribution of new human beta-defensin genes clustered on chromosome 20 in functionally different segments of epididymis Genomics 2003 81 2 175 183 10.1016/S0888-7543(02)00034-4 12620395

[B20] Yamaguchi Y Ouchi Y Antimicrobial peptide defensin: identification of novel isoforms and the characterization of their physiological roles and their significance in the pathogenesis of diseases Proc Jpn Acad Ser B Phys Biol Sci 2012 88 4 152 166 10.2183/pjab.88.152 22498979 PMC3406309

[B21] Garcia JR Identification of a novel, multifunctional beta-defensin (human beta-defensin 3) with specific antimicrobial activity. Its interaction with plasma membranes of Xenopus oocytes and the induction of macrophage chemoattraction Cell Tissue Res 2001 306 2 257 264 10.1007/s004410100433 11702237

[B22] Garcia JR Human beta-defensin 4: a novel inducible peptide with a specific salt-sensitive spectrum of antimicrobial activity FASEB J 2001 15 10 1819 1821 11481241

[B23] Vareille M The airway epithelium: soldier in the fight against respiratory viruses Clin Microbiol Rev 2011 24 1 210 229 10.1128/CMR.00014-10 21233513 PMC3021210

[B24] Kao CY ORFeome-based search of airway epithelial cell-specific novel human beta-defensin genes Am J Respir Cell Mol Biol 2003 29 1 71 80 10.1165/rcmb.2002-0205OC 12600824

[B25] Weinberg A The yin and yang of human Beta-defensins in health and disease Front Immunol 2012 3 294 23060878 10.3389/fimmu.2012.00294 PMC3465815

[B26] Domagala-Kulawik J Effects of cigarette smoke on the lung and systemic immunity J Physiol Pharmacol 2008 59 Suppl 6 19 34 19218630

[B27] Chen X Antimicrobial peptides human beta-defensin (hBD)-3 and hBD-4 activate mast cells and increase skin vascular permeability Eur J Immunol 2007 37 2 434 444 10.1002/eji.200636379 17230440

[B28] Chen L Cigarette smoke enhances beta-defensin 2 expression in rat airways via nuclear factor-kappaB activation Eur Respir J 2010 36 3 638 645 10.1183/09031936.00029409 20150208

[B29] Mahanonda R Cigarette smoke extract modulates human beta-defensin-2 and interleukin-8 expression in human gingival epithelial cells J Periodontal Res 2009 44 4 557 564 10.1111/j.1600-0765.2008.01153.x 19438974

[B30] Semlali A Whole cigarette smoke increased the expression of TLRs, HBDs, and proinflammory cytokines by human gingival epithelial cells through different signaling pathways PLoS One 2012 7 12 e52614 10.1371/journal.pone.0052614 23300722 PMC3532503

[B31] Kulkarni R Cigarette smoke inhibits airway epithelial cell innate immune responses to bacteria Infect Immun 2010 78 5 2146 2152 10.1128/IAI.01410-09 20194598 PMC2863539

[B32] Amer LS Bishop BM van Hoek ML Antimicrobial and antibiofilm activity of cathelicidins and short, synthetic peptides against Francisella Biochem Biophys Res Commun 2010 396 2 246 251 10.1016/j.bbrc.2010.04.073 20399752

[B33] Alekseeva L Inducible expression of beta defensins by human respiratory epithelial cells exposed to Aspergillus fumigatus organisms BMC Microbiol 2009 9 33 10.1186/1471-2180-9-33 19208266 PMC2653505

[B34] Pierson T Proteomic characterization and functional analysis of outer membrane vesicles of Francisella novicida suggests possible role in virulence and use as a vaccine J Proteome Res 2011 10 3 954 967 10.1021/pr1009756 21138299

[B35] Herr C Suppression of pulmonary innate host defence in smokers Thorax 2009 64 2 144 149 10.1136/thx.2008.102681 18852155

[B36] O'Neil DA Expression and regulation of the human beta-defensins hBD-1 and hBD-2 in intestinal epithelium J Immunol 1999 163 12 6718 6724 10586069

[B37] Eckmann L Defence molecules in intestinal innate immunity against bacterial infections Curr Opin Gastroenterol 2005 21 2 147 151 10.1097/01.mog.0000153311.97832.8c 15711205

[B38] Andreeva AV Kutuzov MA Voyno-Yasenetskaya TA Regulation of surfactant secretion in alveolar type II cells Am J Physiol Lung Cell Mol Physiol 2007 293 2 L259 L271 10.1152/ajplung.00112.2007 17496061

[B39] Fehrenbach H Alveolar epithelial type II cell: defender of the alveolus revisited Respir Res 2001 2 1 33 46 10.1186/rr36 11686863 PMC59567

[B40] Hsieh SJ Biomarkers increase detection of active smoking and secondhand smoke exposure in critically ill patients Crit Care Med 2011 39 1 40 45 10.1097/CCM.0b013e3181fa4196 20935560 PMC3148017

[B41] Liu J Relationship between biomarkers of cigarette smoke exposure and biomarkers of inflammation, oxidative stress, and platelet activation in adult cigarette smokers Cancer Epidemiol Biomarkers Prev 2011 20 8 1760 1769 10.1158/1055-9965.EPI-10-0987 21708936

[B42] Morin A Estimation and correlation of cigarette smoke exposure in Canadian smokers as determined by filter analysis and biomarkers of exposure Regul Toxicol Pharmacol 2011 61 3 Suppl S3 S12 20937342 10.1016/j.yrtph.2010.09.020

[B43] Naufal ZS Differential exposure biomarker levels among cigarette smokers and smokeless tobacco consumers in the National Health and Nutrition Examination Survey 1999-2008 Biomarkers 2011 16 3 222 235 10.3109/1354750X.2010.546013 21348778

[B44] Sexton K Proteomic profiling of human respiratory epithelia by iTRAQ reveals biomarkers of exposure and harm by tobacco smoke components Biomarkers 2011 16 7 567 576 10.3109/1354750X.2011.608855 21966894

[B45] Yuchuan H Circulating biomarkers of hazard effects from cigarette smoking Toxicol Ind Health 2011 27 6 531 535 10.1177/0748233710393393 21415095

[B46] Bernert JT Increases in tobacco exposure biomarkers measured in non-smokers exposed to sidestream cigarette smoke under controlled conditions Biomarkers 2009 14 2 82 93 10.1080/13547500902774613 19330586

[B47] Otri AM Variable expression of human Beta defensins 3 and 9 at the human ocular surface in infectious keratitis Invest Ophthalmol Vis Sci 2012 53 2 757 761 10.1167/iovs.11-8467 22232436

[B48] Scharf S Legionella pneumophila induces human beta defensin-3 in pulmonary cells Respir Res 2010 11 93 10.1186/1465-9921-11-93 20615218 PMC2910005

[B49] Gross CA Beta2-agonists promote host defense against bacterial infection in primary human bronchial epithelial cells BMC Pulm Med 2010 10 30 10.1186/1471-2466-10-30 20470412 PMC2881900

[B50] Liao Z Enhanced expression of human beta-defensin 2 in peripheral lungs of patients with chronic obstructive pulmonary disease Peptides 2012 38 350 356 10.1016/j.peptides.2012.09.013 23000304

[B51] Pace E Beta defensin-2 is reduced in central but not in distal airways of smoker COPD patients PLoS One 2012 7 3 e33601 10.1371/journal.pone.0033601 22438960 PMC3306426

[B52] Zhang W Cigarette smoke modulates PGE(2) and host defence against Moraxella catarrhalis infection in human airway epithelial cells Respirology 2011 16 3 508 516 10.1111/j.1440-1843.2010.01920.x 21199162

[B53] Shibata Y Altered expression of antimicrobial molecules in cigarette smoke-exposed emphysematous mice lungs Respirology 2008 13 7 1061 1065 18699806 10.1111/j.1440-1843.2008.01362.x

[B54] Yamin M Cigarette smoke combined with Toll-like receptor 3 signaling triggers exaggerated epithelial regulated upon activation, normal T-cell expressed and secreted/CCL5 expression in chronic rhinosinusitis J Allergy Clin Immunol 2008 122 6 1145 1153 e3 10.1016/j.jaci.2008.09.033 18986692

[B55] Nakamura S Nicotine induces upregulated expression of beta defensin-2 via the p38MAPK pathway in the HaCaT human keratinocyte cell line Med Mol Morphol 2010 43 4 204 210 10.1007/s00795-010-0493-4 21267696

[B56] Lee J Taneja V Vassallo R Cigarette smoking and inflammation: cellular and molecular mechanisms J Dent Res 2012 91 2 142 149 10.1177/0022034511421200 21876032 PMC3261116

[B57] Xu J Xu F Lin Y Cigarette smoke synergizes lipopolysaccharide-induced interleukin-1beta and tumor necrosis factor-alpha secretion from macrophages via substance P-mediated nuclear factor-kappaB activation Am J Respir Cell Mol Biol 2011 44 3 302 308 10.1165/rcmb.2009-0288OC 20160043 PMC3095931

[B58] Kanda N Human beta-defensin-2 enhances IFN-gamma and IL-10 production and suppresses IL-17 production in T cells J Leukoc Biol 2011 89 6 935 944 10.1189/jlb.0111004 21367976

[B59] Winter J Human beta-defensin-1, -2, and -3 exhibit opposite effects on oral squamous cell carcinoma cell proliferation Cancer Invest 29 3 196 201 21280982 10.3109/07357907.2010.543210

[B60] Kreuter A Expression of antimicrobial peptides in different subtypes of cutaneous lupus erythematosus J Am Acad Dermatol 2011 65 1 125 133 10.1016/j.jaad.2010.12.012 21353331

[B61] Shestakova T Immunohistochemical analysis of beta-defensin-2 expression in human lung tumors Exp Oncol 2010 32 4 273 276 21270758

